# Supplementary material for: Fermionic quantum turbulence: Pushing the limits of high-performance computing
Source: PNAS Nexus. 2024 Apr 15;3(5):pgae160. doi: 10.1093/pnasnexus/pgae160 (PMC11070604; doi:10.1093/pnasnexus/pgae160)
Supplement: pgae160_Supplementary_Data [file pgae160_supplementary_data.zip › PNASNEXUS-PNASNEXUS-2024-00448-T-s07.pdf]

Supplementary Material for: Fermionic Quantum Turbulence: Pushing the Limits of High-Performance Computing

Gabriel Wlazłowski,[1,2,*] Michael McNeil Forbes,[3,2,†] Saptarshi Rajan Sarkar,[3,‡] Andreas Marek,[4,§] and Maciej Szpindler[5,¶]

[1]*Faculty of Physics, Warsaw University of Technology, Ulica Koszykowa 75, 00-662 Warsaw, Poland*
[2]*Department of Physics, University of Washington, Seattle, Washington 98195–1560, USA*
[3]*Department of Physics and Astronomy, Washington State University, Pullman, WA 99164, USA*
[4]*Max Planck Computing and Data Facility, Garching D-85747, Germany*
[5]*Academic Computer Centre CYFRONET, University of Science and Technology, Nawojki 11, 30-950 Cracow, Poland*

RAW DATA

We provide raw data through Zenodo repository [1]. The datasets include:

1. dataset with results for BCS run ($ak_F = -1.8$) obtained with TDSLDA method (`bcs` folder).

2. results of static calculations for single vortex line for BCS run ($ak_F = -1.8$) obtained with SLDA method (`bcs_static_vortex` folder).

3. dataset with results for UFG run ($ak_F = \infty$) obtained with TDSLDA method (`ufg` folder).

4. dataset with results for UFG run ($ak_F = \infty$) obtained with GPE method. The results are provided for two dissipation coefficients $\eta = 0.01$ and 0.08 (`ufg_gpe` folder)

The files contain the following information:

- Time evolution of the density $n(\boldsymbol{r}, t)$, the current $\boldsymbol{j}(\boldsymbol{r}, t)$ and the order parameter $\Delta(\boldsymbol{r}, t)$.
- Positions of extracted vortex lines from time-dependent runs.
- Reproducibility packs for TDSLDA runs. They contain the full information about the settings and the computation process.
- Static solutions for the single vortex in the BCS regime ($ak_F = -1.8$).

In the repository, there is `README.md` file that provides information about used data formats. There are also provided example scripts/codes demonstrating how to read data in python and C.

LIST OF MOVIES

Below, we provide the list of accompanying movies (in mp4 format). 3D views were created by VisIt software [2]. The visualizations presented the volume distribution of the order parameter and lines indicating the vortex cores' location. All movies are also accessible on YouTube.

Supplementary movie 1: Run for the unitary Fermi gas ($ak_F = \infty$) by means of TDSLDA.
YouTube: youtu.be/hOLPmPVQ4xo

Supplementary movie 2: Run for the BCS regime ($ak_F = -1.8$) by means of TDSLDA.
YouTube: youtu.be/OpIDOPdaPKM

Supplementary movie 3: Run for the unitary Fermi gas ($ak_F = \infty$) by means of GPE, with the dissipation coefficient $\eta = 0.01$.
YouTube: youtu.be/3Dux6PHD4e4

Supplementary movie 4: Run for the unitary Fermi gas ($ak_F = \infty$) by means of GPE, with the dissipation coefficient $\eta = 0.081$.
YouTube: youtu.be/7FXl-NIAq20

Supplementary movie 5: Dynamics of vortex lines and their local effective temperatures for the unitary Fermi gas ($ak_F = \infty$).
YouTube: youtu.be/Wd_gEUZcbu4

Supplementary movie 6: Dynamics of vortex lines and their local effective temperatures for the BCS regime ($ak_F = -1.8$).
YouTube: youtu.be/zGSaVhT3e74

THERMOMETER CALIBRATION CURVE

In Fig. 1, we provide the relation between vortex core density (normalized to the bulk density) and gas temperature. These curves were used for assign the local temperature of vortex cores, as shown in movies `Supplementary movie 5` and `Supplementary movie 6`.

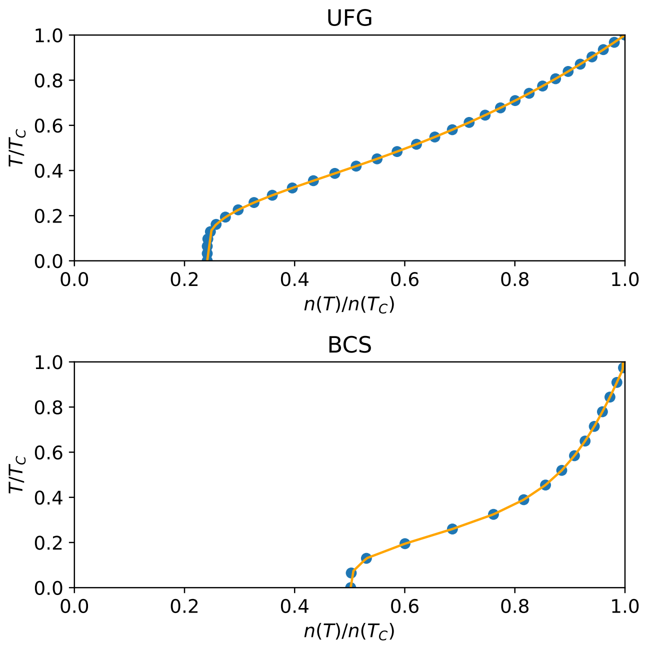

FIG. 1. The relation between the vortex core density and the gas temperature for UFG (top) and BCS (bottom) regimes. These calibration curves were used to assign the local effective temperatures, as presented in Fig. 8.

REFERENCES

* gabriel.wlazlowski@pw.edu.pl
† m.forbes@wsu.edu
‡ saptarshi.sarkar@wsu.edu
§ andreas.marek@mpcdf.mpg.de
¶ m.szpindler@cyfronet.pl

[1] Gabriel Wlazłowski, Michael McNeil Forbes, Saptarshi Rajan Sarkar, Andreas Marek, and Maciej Szpindler, "Quantum turbulence in superfluid Fermi gas: results of numerical simulation," (2023), available at doi.org/10.5281/zenodo.8355244.

[2] Hank Childs, Eric Brugger, Brad Whitlock, Jeremy Meredith, Sean Ahern, David Pugmire, Kathleen Biagas, Mark Miller, Cyrus Harrison, Gunther H. Weber, Hari Krishnan, Thomas Fogal, Allen Sanderson, Christoph Garth, E. Wes Bethel, David Camp, Oliver Rübel, Marc Durant, Jean M. Favre, and Paul Navrátil, "VisIt: An end-user tool for visualizing and analyzing very large data," in *High Performance Visualization–Enabling Extreme-Scale Scientific Insight* (2012) pp. 357–372.
